# Improving care for individuals with gender incongruence: Establishing a multidisciplinary approach in Italy

**DOI:** 10.1007/s40618-025-02580-x

**Published:** 2025-06-06

**Authors:** Alberto Scala, Chiara Ceolin, Marina Miscioscia, Daniela Basso, Elena Campello, Valentina Camozzi, Annamaria Cattelan, Michela Gatta, Sandro Giannini, Massimo Iafrate, Giulia Musso, Paolo Meneguzzo, Giancarlo Ottaviano, Rossella Perilli, Roberta Rosin, Ilaria Ruzza, Carlo Saccardi, Lolita Sasset, Giuseppe Sergi, Benedetta Tascini, Tommaso Vezzaro, Fabrizio Vianello, Paolo Simioni, Alberto Ferlin, Andrea Garolla, On behalf of the Interdisciplinary Group for Gender Incongruence (GIIG)

**Affiliations:** 1 Unit of Andrology and Reproductive Medicine, Department of Medicine (DIMED), University of Padua, Padua, Italy; 2 Regional Reference Center for Gender Incongruence (CRRIG), University Hospital of Padua, Veneto, Italy; 3 Geriatrics Division, Department of Medicine (DIMED), University of Padua, Padua, Italy; 4 Department of Neurobiology, Care Sciences and Society, Aging Research Center, Karolinska Institutet and Stockholm University, Stockholm, Sweden; 5 Department of Developmental Psychology and Socialization, University of Padua, Padua, Italy; 6 Child and Adolescent Neuropsychiatric Unit, Department of Women’S and Children’S Health, University Hospital of Padua, Padua, Italy; 7 Laboratory Medicine, Department of Medicine (DIMED), University of Padua, Padua, Italy; 8 Haemorrhagic and Thrombotic Diseases Unit, Department of Medicine (DIMED), University of Padua, Padua, Italy; 9 Department of Medicine, Medical Clinic 1, University of Padova, Padua, Italy; 10 Unit of Endocrinology, Department of Systems Medicine (DIDAS), University Hospital of Padua, Padua, Italy; 11 Infectious Disease Unit, Department of Systemic Medicine (DIDAS), University Hospital of Padua, Padua, Italy; 12 Department of Surgical, Oncological and Gastroenterological Sciences (DISCOG), Urologic Clinic, University Hospital of Padua, Padua, Italy; 13 Department of Neurosciences, University of Padua, Padua, Italy; 14 Department of Neurosciences, Otolaryngology Section, University of Padua, Padua, Italy; 15 Innovation and Organizational Development Office, University Hospital of Padua, Padua, Italy; 16 CON-TE-STARE Sportello Accoglienza Transgender– Centro ONIG, Padua, Italy; 17 SAT-Pink– Servizio Accoglienza Trans*, Padua, Italy; 18 Department of Gynaecology and Obstetrics, Padova University Hospital, University of Padua, Padua, Italy; 19 Hematology and Clinical Immunology Unit, Department of Medicine, University of Padua, Padua, Italy; 20 Unit of Andrology and Reproductive Medicine, Department of Medicine (DIMED), University of Padua, Via Giustiniani, 2, 35121 Padua, Italy

**Keywords:** Transgender, Gender-affirming care, Health, Person-centered care, Multidisciplinary team

## Abstract

**Purpose:**

To present a multidisciplinary care model designed to provide personalized gender-affirming care and assess general health for transgender and gender-diverse (TGD) individuals.

**Methods:**

Drawing from our experience in a tertiary center in Padua (Italy), the Interdisciplinary Group for Gender Incongruence (GIIG) model employs a multidisciplinary approach to provide diverse gender-affirmation services. Mental health support, gender-affirming medical and surgical treatments (GAMST), screening programs, and regular follow-up ensure treatment safety and efficacy. The GIIG model promotes collaboration among specialists, primary health services, and LGBTQ + associations. Furthermore, it advocates for training healthcare professionals and raising awareness in the population.

**Results:**

The GIIG involves mental health professionals, endocrinologists, surgeons (plastic, urological, and gynaecological), voice specialists, internists, and associations. Associations serve as a point of reference for the community and offer psychological and legal services. Initial contact at the Center is made with MHP, who provide a safe space to explore gender identity, receive information, and support mental well-being. Endocrinologists prescribe hormone therapy and monitor its potential risks and overall health. Surgical interventions include chest, genital, and laryngeal surgery. Internists and other specialists assess osteo-muscular, hemo-coagulative, oncological, and infectious risks.

**Conclusions:**

Our experience emphasizes the need for personalized care tailored to individuals’ desires while ensuring the safety of gender-affirming treatments. By adhering to the Standards of Care and offering comprehensive services, our center aims to serve as a model for modern transgender care.

## Introduction

In recent years, there has been a notable increase in the visibility of transgender and gender-diverse (TGD) people in public spaces, paralleled by a rising demand for gender-affirming care. Population-based surveys indicate that TGD individuals comprise approximately 0.3–0.5% of the adult population, with a higher prevalence among adolescents (1.2–2.7%) [1, 2]. Many TGD individuals seek gender-affirming medical and surgical treatments (GAMST) to align their physical appearance with their gender identity [3].

Gender-affirming programs typically start with an assessment by a mental health professional to explore one’s gender identity, assess capacity for informed consent and offers psychological support before proceeding with medical interventions [1, 4]. After a general health assessment, gender-affirming hormone therapy (GAHT) is initiated to induce body masculinization or feminization. Follow-up visits monitor its effects and potential risks [1, 4]. Fertility preservation options should be explored before GAMST [1, 3, 5, 6].

Approximately 25–35% of TGD individuals opt for gender-affirming surgery (GAS), which may include “top surgery” and “bottom surgery” procedures [7]. In Italy, Law No. 164/1982 requires court authorization both to legally change one’s name and gender on official documents and to undergo GAS [8]. In this context, LGBTQIA + organizations play a crucial role in providing legal support and helping individuals navigate the bureaucratic system. Additional gender-affirming interventions, such as laryngoplasty for voice feminization, may also be considered [9]. Collaboration between tertiary care centers, local health services, and advocacy associations is essential to ensure comprehensive care and support throughout the gender-affirmation process.

However, even within medical environments, TGD individuals too often experience significant health disparities as a result of gender minority stress (GMS), resulting in elevated rates of depression, anxiety, bone frailty, and cardiovascular events [10, 11]. Improving healthcare access and quality, especially from a young age, is crucial to mitigate the psycho-physical effects of GMS. Multidisciplinary gender-affirming care, as endorsed by international health organizations, involves professionals such as MPH, endocrinologists, and surgeons, facilitating informed decision-making and empowering TGD individuals to actively engage in the process [1].

Given the good results derived from the organization of culturally responsive and clinically competent care in different settings, we established a multidisciplinary program to improve health care for TGD people in Italy.

## Methods

### Project design

From 2021, the Interdisciplinary Group for Gender Incongruence (GIIG) has been established at the University Hospital of Padua (Italy), bringing together healthcare professionals involved in TGD care. In 2023, the region of Veneto officially designated Padua as the Regional Reference Center for Gender Incongruence (CRRIG).

**Fig. 1:**
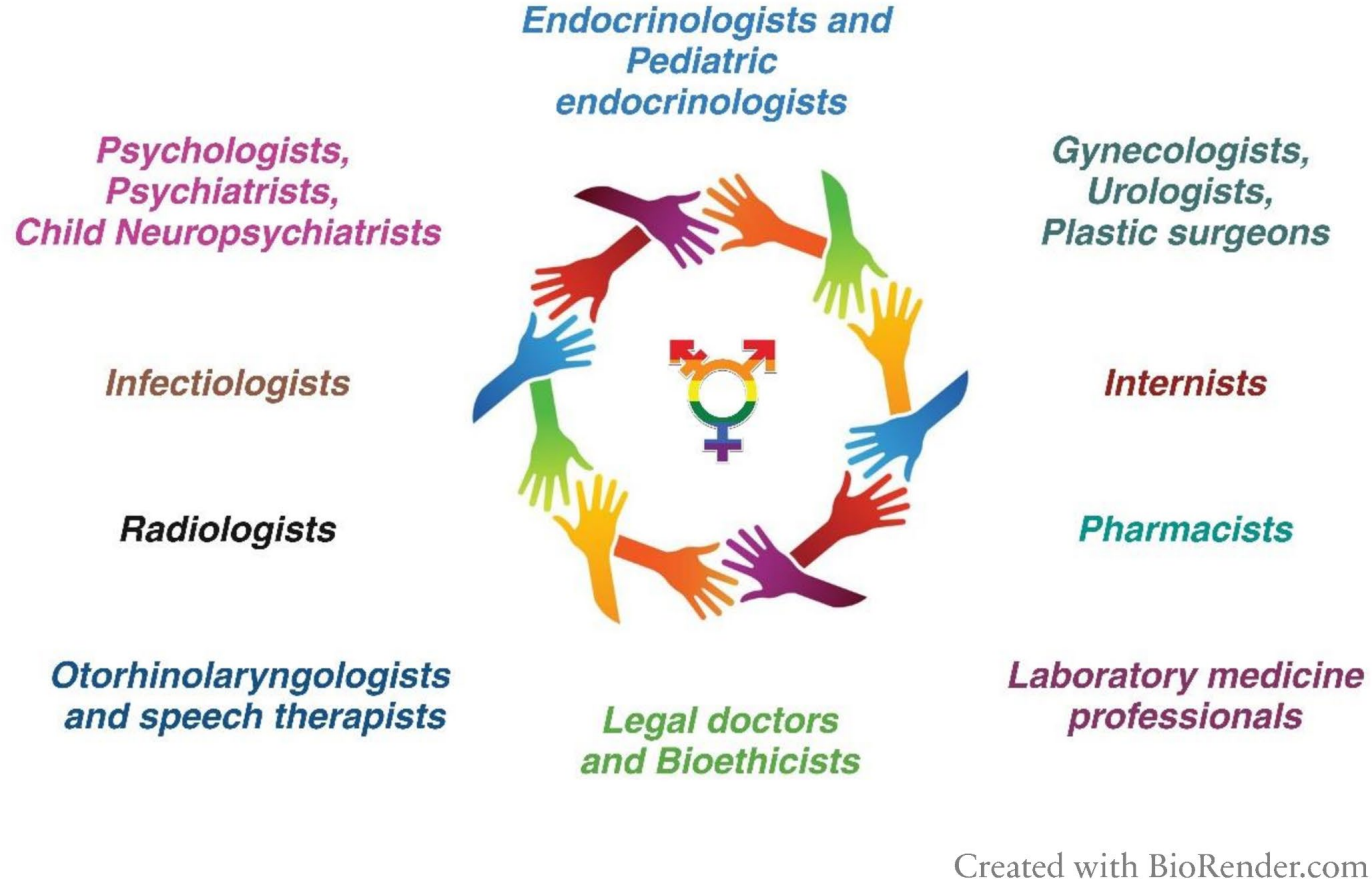
The Interdisciplinary Group for Gender Incongruence (GIIG)

The drafted GIIG model, thanks to the active involvement of a panel of experts, outlines a reference operational framework that describes a fluid and articulated process, identifying the procedural steps: initial contact and booking of provided services; psychological support; personalized GAMST program; and engagement with the community and associations. These steps, guided by international Standards of Care, enable our Center to achieve better care outcomes. Specifically, all the figures involved in the interdisciplinary group are shown in Fig. 1.

### Purpose

In line with the latest guidelines, the CRRIG is dedicated to providing comprehensive care to individuals seeking GAMST [1, 3, 4]. The center prioritizes a multidisciplinary approach, ensuring a diverse array of services to meet the unique needs of each TGD person. Moreover, the center emphasizes holistic well-being, offering mental health support, screening programs, and regular follow-up visits to ensure the safety and efficacy of treatments.

The adoption of the multidisciplinary GIIG model is intended to yield various positive outcomes, including:

- Ensuring a comfortable healthcare environment for TGD individuals.
- Provide information regarding gender affirmation process.
- Create a collaborative team of specialists and organize periodic multidisciplinary meetings.
- Training healthcare personnel and promote awareness in the general population.
- Establishing a territorial assistance network that involves associations, general practitioners, secondary and tertiary center.

## Results

### Phases

In this section, we present a brief overview of each phase of gender-affirmative care provided by the GIIG team (Table 1).

**Table 1:** Macrophases and different specialized skills in the interdisciplinary group for gender incongruence (GIIG) model

Macrophase	Activities	Description	Personnel
*Mental health*	*Child and adolescent neuropsychiatric evaluation*	• Semi-structured and free interviews • Questionnaires are administered to integrate and complete clinical impressions	Child Neuropsychiatrist Psychologist
	*Adult psychiatric and psychological evaluation*	• Semi-structured interview to gather comprehensive data about people • Questionnaires are administered to evaluate constructs such as body image dissatisfaction, psychiatric symptoms, and dysfunctional behaviors	Psychologist Psychiatrist
*Endocrinology*	*Endocrinological evaluation; coordination and referral to other specialists*	• First medical contact • General health screening • GAHT prescription • Referral to other specialists	Endocrinologist
*Surgery*	Gender-affirming surgery Gynecological care	• For AMAB individuals, breast augmentation and orchiectomy may be offered, potentially followed by vaginoplasty • For AFAB TGD individuals, chest masculinization, hysterectomy, and/or salpingo-oophorectomy may be offered, potentially followed by metoidioplasty or phalloplasty • A gynecological service dedicated to TGD individuals is available at our hospital	Urologist Gynecologist Plastic Surgeon
*Voice and communication*	*Voice training*	• Speech and language treatments, such as conservative voice therapy or with endoscopic glottoplasty	Speech therapist
	*Voice surgery*	• Vocal cord surgery • Surgical decrease of the laryngeal prominence	Otolaryngologist
*General health*	*Bone health*	• Bone densitometry and phosphor-calcic metabolism measurement • Assessment of bone health and fracture risk	Internist Endocrinologist
	*Hematological risk evaluation*	• Screening for hereditary thrombophilia • Counselling with a couagulation specialist in case of VTE or thrombophilia • Hematological evaluation in case of severe androgen-induced erythrocytosis	Internist Hematologist
	*Infective risk management*	• Testing, information, and treatment of HIV, HPV and other STIs • Offering HBV, HAV, and HPV vaccination	Infectiologist
*Associations*	*SAT-Pink*,* Con-Te-Stare*	• Ongoing training for all members and cultural sessions to enhance awareness of gender issues • Promotion of destigmatization of trans* and non-binary experiences • Support in navigating bureaucratic and legislative constraints • Other services provided by professionals (psychologists, lawyers, etc.)	Activists and volunteers Professionals

AMAB = Assigned Males At Birth; AFAB = Assigned Females At Birth; GAHT = Gender Affirming Hormone Therapy; VTE: venous thromboembolism; HIV = Human Immunodeficiency Virus; HPV = Human Papilloma Virus; STI = Sexually Transmitted Infection; HBV = Hepatitis B Virus; HAV = Hepatitis A Virus

### Mental health

**Assessment for TGD individual requiring GAMST**. In Italy, access to GAMST requires a diagnosis of gender incongruence (GI) or gender dysphoria (GD) according to the criteria outlined in the International Classification of Diseases, 11th Revision (ICD-11) and the Diagnostic and Statistical Manual of Mental Disorders, 5th edition (DSM-5), respectively. This involves the collaboration of psychologists, psychiatrists, and child neuropsychiatrists [1].

Assessments, which include semi-structured interviews and the use of standardized questionnaires, are crucial for guiding TGD individuals through the gender affirmation process [12]. In child and adolescent clinical settings, assessments involve semi-structured and open interviews, along with the use of standardized questionnaires, to gather data on personal and family history, evaluate psychological functioning, and identify any distress in mental well-being [13]. The frequency and duration of sessions are personalized based on individual needs.

The success of the gender affirmation process relies on mental well-being, necessitating awareness of the higher prevalence of psychiatric conditions among TGD individuals (e.g., depression, anxiety, suicidal thoughts) and attention to concerns related to body image. Consultations and treatment are tailored to the specific needs of each person, providing support in their journey of self-discovery and promoting personal resilience [12, 14, 15].

**Support for mental well-being.** Throughout the assessment process, our team provides support and strengthens the individual’s mental health while reinforcing their resilience. We also assist in addressing stigma and minority stress-related conditions, and help individuals find affirming spaces and support within social communities [16].

In child and adolescent clinical settings, our aim is to inform, support, and involve the family in the process, recognizing that parental support significantly reduces psychiatric symptoms and life dissatisfaction among TGD youth [14]. Psychological interventions are available for both adolescents and adults who are TGD, targeting common concerns related to GD, the coming out process, career aspirations, and parenthood desire.

While psychotherapy is not obligatory to access GAMST, it can be beneficial for some individuals to explore their identity, enhance self-acceptance, and build resilience, particularly in challenging environments [17].

### Endocrinology

The endocrinologist, in addition to prescribing hormone therapy, serves as the primary medical contact throughout the gender affirmation journey. They assess overall health, elaborate individualized treatment plans, and coordinate referrals to other specialists as necessary. During the initial consultation, patients discuss their desired physical changes and treatment expectations, in order to customize GAHT [1, 3, 4]. Patients receive comprehensive education on available treatments, expected effects, and potential risks [1, 3, 4]. Collaborating with graphic artist *Daigoro* (Nicky Bonetto), we created informative materials on GAHT that are both intuitive and evidence-based.

GAHT is typically prescribed during the first follow-up visit, with the endocrinologist reviewing prescribed exams. In the absence of significant health issues, the endocrinologist develops a treatment plan [1, 3, 4]. The prescription from a specialized center grants cost-free access to GAHT [18]. Referrals to other specialists are made for significant health issues to ensure the safety of GAHT. Follow-up appointments are scheduled every three months for the first year, and then once or twice a year thereafter [1, 3, 4].

#### Pediatric endocrinology

The onset of secondary sexual characteristics during puberty can be a significant source of distress for adolescents with gender dysphoria [19, 20]. A multidisciplinary team—including a Child Neuropsychiatrist, Psychologist, Pediatrician, Endocrinologist, and Bioethicist—assesses each case individually. The first-line intervention consists of psychological and neuropsychiatric support to help adolescents navigate distress related to gender variance [1, 21]. The pediatrician evaluates the general health and auxological progression of children and young adolescents. Gender-affirming hormone therapy may begin around age 16, with continuous psychological support throughout the process [1, 3].

According to the Standards of Care, TGD adolescents experiencing significant distress due to gender dysphoria may benefit from puberty suppression [1]. GnRH analogue therapy is a reversible intervention that can be considered after Tanner stage 2 and serves a dual purpose: first, by halting the progression of secondary sexual characteristics, it aims to protect psychological well-being and reduce the risk of suicide and depression; second, it provides adolescents time to explore their gender identity before irreversible treatments [1, 21, 22].

A working group at our hospital has been assessing access to triptorelin. However, the regulatory framework for its implementation remains uncertain due to the recent publication of a document from the National Bioethics Committee, which stipulates that triptorelin should only be utilized in experimental protocols, although the medication is provided free of charge under the Italian Medicines Agency (AIFA) Directive, 2019 [23, 24].

#### Fertility preservation

Due to the significant impact of GAHT on reproductive function, including effects on ovulation and spermatogenesis, and the uncertainty regarding reversibility upon therapy cessation, fertility preservation and contraception discussions are extensively held before initiating GAMST [5, 6]. Our Center currently lacks cryopreservation services, as this service is not offered by the Regional Health System in the context of gender-affirming treatments, necessitating individuals to seek private facilities. However, there is an increasing demand to establish cryopreservation services within the Nation Health Service to address potential fertility implications for TGD individuals undergoing GAMST.

### Surgery

Plastic Surgeons, Gynecologists, and Urologists are the main surgical figures involved in GAS. These procedures encompass ‘top surgery’ (e.g., mastectomy, breast augmentation) and ‘bottom surgery’ (e.g., gonadectomy, vaginoplasty, phalloplasty) [1]. Studies consistently report high rates of satisfaction related to GAS, including improvements in quality of life, body image, and reduction in gender dysphoria, while rates of regret remain low [25]. In Italy, legal authorization is required before proceeding with GAS [8]. Prior to surgery, each case undergoes a multidisciplinary meeting with surgeons, mental health professionals, endocrinologists, and eventually internists, to discuss the general health status, mental well-being, fertility preservation options, and surgical techniques.

**Plastic surgery**. The plastic surgeon plays a crucial role in performing top surgery. Bilateral mastectomy, also called chest masculinization, is sought by assigned female at birth (AFAB) individuals to achieve a flat chest, typically using techniques like double incision with free nipple graft, or periareolar incision [26]. Conversely, many AFAB individuals opt for mammoplasty for breast enhancement, since full breast development occurs in less than 20% of cases with estrogen therapy [27]. Breast implant placement is often accompanied by nipple resizing for a natural look [28].

These procedures are aims to enhance body confidence, with research indicating high satisfaction rates and complication rates similar to cisgender patients [29, 30].

**Gynecological surgery**. Gynecologists can perform procedures such as hysterectomy and bilateral salpingoophorectomy (BSO) for AFAB individuals, minimizing surgical trauma through laparoscopy or transvaginal approaches [1]. Surgical procedures like hysterectomy and BSO are typically straightforward, but counseling should extend beyond technical aspects [31]. Discussion about the irreversible loss of reproductive function and comprehensive fertility counseling is mandatory before genital surgery [5, 6].

**Urological surgery**. In the context of GAS, urological procedures aim at modifying primary sexual organs. For assigned male at birth (AMAB) individuals, bilateral orchiectomy may be proposed to lower endogenous androgen levels [32].

**Reconstructive genital surgery**. Our center currently performs orchiectomy and hysteroannessiectomy, referring patients for reconstructive genital surgeries. Our goal is to expand our capabilities to offer these procedures directly.

Specifically, AMAB individuals may choose to undergo vaginoplasty [33]. AFAB individuals may opt for phalloplasty, which can include penile prosthesis insertion, or metoidioplasty, a procedure that creates a small neophallus from an enlarged clitoris [33, 34].

### Voice and communication

Speech and language therapists play an important role in helping TGD individuals overcome voice dysphoria, which is crucial for accepting their new identity. They assess and address voice issues subjectively and objectively, especially before vocal cord surgery. Voice feminization often involves conservative speech therapy to adjust pitch and resonance and find their unique voice [9]. The therapeutic process may include several goals: voice hygiene (maintaining vocal cord health and preventing injury), pitch modification (adjusting voice frequency), intonation (shaping vocal tone and quality), volume control, speech rhythm and rate, and articulation. Additionally, speech therapists teach the importance of non-verbal communication, including gestures and facial expressions, as well as pragmatic language skills, such as adjusting voice for different social contexts, expressing emotions effectively, and navigating communication across languages and cultural situations [35]. Currently, there are no evidence-based guidelines for vocal gender affirmation. However, in our practice, therapists utilize resonant voice therapy and semi-occluded vocal tract exercises, both of which have been extensively studied and validated [36, 37].

Otolaryngologist can aid voice feminization through laryngeal surgery, such as endoscopic glottoplasty [9, 38]. Sometimes vocal cord surgery is associated with laryngeal shave or septorhinoplasty [39].

### General health

**Bone metabolism**. GAHT therapy induces significant changes in body composition, with TGD AMAB individuals experiencing increased fat mass and decreased muscle mass, while AFAB individuals exhibit increased muscle mass and strength [40]. Studies indicate that AMAB individuals have lower bone mineral density (BMD) compared to cisgender peers, prompting a baseline evaluation of bone density and phosphor-calcium metabolism [41, 42]. Dual Energy X-ray Absorptiometry (DXA) is used to assess BMD at proximal femur and lumbar spine. Patients with lower BMD are identified early for intervention, including lifestyle modifications, vitamin D and calcium supplementation, or pharmacological therapies (e.g., bisphosphonates) [42].

**Erythrocytosis**,** thrombophilia and cardiovascular risk**. Hormone therapy influences cardiovascular risk factors like body weight, blood pressure, and lipid profile [10]. Furthermore, estrogen therapy induces a pro-coagulant state that leads to an increased rate of thromboembolic events compared to the cisgender population, while testosterone-induced erythrocytosis is a common adverse effect in AFAB individuals [43–45]. Before starting GAHT, a comprehensive evaluation is performed, including blood pressure, weight, haematocrit, glucose and lipid profile, and screening for congenital thrombophilia in AMAB individuals. Consultation with haematologists and experts of coagulation is sought for individuals with congenital thrombophilia or androgen-induced erythrocytosis. In these cases, therapy adjustment is made in order to reduce the hematocrit and pro-thrombotic risk.

**Gynaecological evaluation**. TGD individuals may avoid medical care due to concerns about discrimination or disrespectful behavior from healthcare providers [46]. This avoidance is particularly pronounced for gender-specific procedures, such as gynecological evaluations and screening programs for cervical and breast cancer [47, 48].

However, gynecology is not exclusively for cisgender women. Transgender and non-binary individuals also deserve inclusive, culturally sensitive care [49]. To address this need, a gynecological service dedicated to TGD individuals was established, offering counseling on menstrual suppression, contraception, reproductive and sexual health, genitourinary symptoms, and oncological screenings. Additionally, gynaecological follow-up is essential for transgender women who have undergone vaginoplasty.

**Infectious risk management**. Sexually transmitted infections (STI) pose a global health challenge, often presenting asymptomatic and contributing to inadvertent transmission during unprotected sex. If untreated, STI can lead to severe complications, including infertility and increased HIV transmission [50]. Population studies have shown that TGD individuals face a significant risk of HIV and other STI [51].

Screening for STI is routinely offered to TGD individuals who express interest in being tested, alongside education on STI prevention, vaccination, testing, and treatment. The infectious disease unit guarantees medical care for patients diagnosed with STI.

**Laboratory medicine**. Biochemical testing is crucial for monitoring the effects and potential side effects of GAMST. This includes assessing sexual hormone levels, blood count, liver and kidney function, metabolic profile, phosphor-calcic metabolism, and bone turnover markers [52]. Moreover, laboratory professionals are working to create reference ranges suitable for TGD individuals undergoing GAHT, aiming to overcome limitations of sex-based ranges (e.g. creatinine).

**Pharmacy**. In accordance with *AIFA Directive 2020*, GAHT is fully covered by the National Health Service for TGD individuals receiving care from a multidisciplinary team in a specialized gender clinic [18]. With a treatment plan prescribed by an endocrinologist, they can obtain their medications from hospital pharmacies in their area of residence.

**Bioethics and legal assistance**. The bioethicist is an essential member of the multidisciplinary team, as required by AIFA directives [18, 23]. They support the GIIG in multidisciplinary meetings for complex cases, contribute to the development of clinical care pathways, and provide guidance in drafting clinical research protocols.

A forensic physician is consulted for legal interpretations related to gender affirmation. For example, they provide guidance in cases where TGD individuals have changed their documents abroad through an administrative process but require GAS in Italy. Additionally, legal assistance is available through associations that have agreements with lawyers with experience in legal name and gender reassignment.

### Associations

*SAT Pink Aps* provides comprehensive support for trans*, non-binary, and gender non-conforming individuals in Veneto and neighboring regions. With a team of volunteers and professionals, including TGD individuals, SAT Pink offers up-to-date information, ongoing training, and advocacy for destigmatization and self-determination. They assist in navigating bureaucratic and legislative hurdles while respecting individual paths without imposing mandatory phases. SAT Pink does not adhere to conventional gender affirmation pathways and refrains from imposing mandatory phases on those seeking assistance.

*Con-Te-Stare Sportello Attivo Transgender* aims to connect associations and local entities supporting TGD people, guided by the principles of the Italian National Observatory on Gender Identity (ONIG). Offering psychological, endocrinological, legal, naturopathic, and aesthetic support, the association promotes self-determination, non-discrimination, and cultural openness. It collaborates with the University of Padua, conducts awareness-raising activities, and hosts groups for seniors and parents of children undergoing gender-affirming pathways.

## Discussion

In this article, we presented the first description of a multidisciplinary model of care for TGD individuals in Italy, focusing on both young and adult individuals. The emphasis is on the need to provide personalized and tailored care to meet individual needs. Attention is directed not only towards gender-affirming treatments, but also to potential medical complications and overall health.

Despite destigmatization efforts by organizations like the World Health Organization and the American Psychological Association, access to GAMST still relies heavily on mental health diagnoses and various “gatekeepers” [53]. Initial editions of the Standards of Care mandated individuals seeking GAMST to live in their chosen gender for specific durations, alongside diagnostic criteria [54]. The 8th version maintains the diagnosis requirement, while promoting a shift towards a more person-centered approach, based on informed consent and shared decision-making [1]. Despite positive impacts on reducing stigma and improving healthcare access for the TGD community, challenges persist, particularly concerning the pathologizing approach of diagnosis and the role of clinicians as gatekeepers [54, 55].

Worldwide, efforts to optimize transgender care have led to the emergence of new multidisciplinary centers, often providing support to both TGD individuals and their families [56, 57]. While these centers typically include professionals like physicians, nurses, psychologists, and social workers, their organization varies. Unfortunately, there is a lack of standardized models for optimal multidisciplinary center configuration. For instance, *Sotiros et al.* propose a centralized care model where TGD people meet various providers in one visit [58]. *Chen et al.* propose a model specifically for TGD children and adolescents, with simultaneous consultations with medical and psychological professionals to streamline care and reduce repetition [59]. With increasing numbers of gender-variant children and adolescents, specialized centers for TGD youth are emerging, including in Italy [56, 57]. However, the distribution of multidisciplinary centers across the national territory is not uniform.

The establishment of a multidisciplinary team was a recent milestone for our hospital. Until 2020, transgender care was limited to the endocrinological service. The formation of the multidisciplinary GIIG team marked a turning point, allowing us to provide comprehensive healthcare services, including psychological support, medical and surgical gender-affirming treatments, and measures to improve overall health. The team benefits from the enthusiasm of specialists who collaborate to cultivate a welcoming and inclusive healthcare environment. Periodic multidisciplinary meetings are organized to discuss patients’ gender-affirming programs, especially prior to surgery or in case of adolescents. Our partnership with advocacy groups provides crucial resources for navigating gender affirmation services, including legal assistance, and offers a supportive community for TGD people.

Despite these advancements, we acknowledge that several objectives remain ongoing challenges for our Center. Currently, we lack the expertise to provide reconstructive bottom surgery, and fertility preservation services are limited to oncological patients in the public health system. Future goals include acquiring the skills to perform vaginoplasty, phalloplasty, and metoidioplasty in-house. We also aim to strengthen collaboration with primary healthcare facilities in our region to facilitate access to GAMST. Additionally, we plan to organize training sessions for healthcare professionals and informative events for the general population to increase awareness on gender identity issues.

## Conclusions

The increasing demand for gender-affirming services emphasizes the need for specialized tertiary centers dedicated to transgender care. Multidisciplinary models, like ours and others globally, represent a significant advancement in meeting the diverse needs of TGD individuals across different age groups. Through multidisciplinary assessment and a wide range of services, our experience in Padua highlights the importance of delivering personalized care, addressing both GAMST and overall health.

We hope that our model can serve as an example for the establishment of new multidisciplinary services for gender-affirming care, contributing to the development of a comprehensive network of services for transgender individuals.
